# Variations in Fatty Acids Affected Their Derivative Volatiles during Tieguanyin Tea Processing

**DOI:** 10.3390/foods11111563

**Published:** 2022-05-26

**Authors:** Li Guo, Mingjie Chen, Yaling Guo, Zhi Lin

**Affiliations:** 1 Tea Research Institute, Chinese Academy of Agricultural Sciences, Hangzhou 310008, China; guoli@tricaas.com; 2 Henan Key Laboratory of Tea Plant Biology, College of Life Sciences, Xinyang Normal University, Xinyang 464000, China; 3 College of Horticulture, Fujian Agriculture and Forestry University, Fuzhou 350002, China; yaling7819@126.com

**Keywords:** oolong tea, Tieguanyin tea, fatty acids, fatty acid-derived volatile (FADV), aroma, volatile compound

## Abstract

Fatty acids (FAs) are important precursors of oolong tea volatile substances, and their famous derivatives have been shown to be the key aroma components. However, the relationship between fatty acids and their derivatives during oolong tea production remains unclear. In this study, fresh Tieguanyin leaves were manufactured into oolong tea and green tea (control), and fatty acids and fatty acid-derived volatiles (FADV) were extracted from processed samples by the sulfuric acid–methanol method and solvent-assisted flavor evaporation (SAFE), respectively. The results showed that unsaturated fatty acids were more abundant than saturated fatty acids in fresh leaves and decreased significantly during tea making. Relative to that in green tea, fatty acids showed larger variations in oolong tea, especially at the green-making stage. Unlike fatty acids, the FADV content first increased and then decreased. During oolong tea manufacture, FADV contents were significantly and negatively correlated with total fatty acids; during the green-making stage, methyl jasmonate (MeJA) content was significantly and negatively correlated with abundant fatty acids except steric acid. Our data suggest that the aroma quality of oolong tea can be improved by manipulating fatty acid transformation.

## 1. Introduction

Oolong tea is known for its natural fruity and floral aroma among the six kinds of Chinese tea, including Tieguanyin tea, Dahongpao tea, Fenghuang Dancong tea, etc. Tieguanyin tea, which originated from Fujian Province, has a unique orchid note and is highly favored by consumers, so its aroma chemistry has become a prominent research topic. The odor characteristics of Tieguanyin tea differ with their production areas [1], grades [2], and aroma types [3], and the main odorant compounds were identified by Lin et al. [4] and Ji et al. [5]. Zhu et al. [6] found that the number of aroma-active compounds in Tieguanyin tea was less than that in Dahongpao tea and Fenghuang Dancong tea, and the aroma intensities of linalool and (E)-2-heptenal were relatively high; however, the contents of trans-nerolidol, indole, α-farnesene, etc., were higher [5]. In addition to these key volatiles, MeJA and jasmine lactone, two degradation products from fatty acids, were also identified [7,8]. MeJA and jasmine lactone have a floral aroma, have been shown to contribute to the aroma quality of Tieguanyin tea, and are regarded as the marker compounds of multiple stresses [9]. The production of fatty acid metabolites during the green-making stage of oolong tea has been shown to depend on the levels of turnover. In addition to jasmonate compounds [10,11], other FADV with green note, such as cis-3-hexenol, trans-2-hexenal, and hexyl hexanoate, were also likely to be formed and retained in the tea; thus, it is important to regulate fatty acid degradation during manufacturing and to optimize the proportion of FADV.

Green-making technology is utilized first for oolong tea and becomes the key mechanism of floral compound formation. According to the traditional method, green-making technology is divided into four or five steps (four to five turnovers). The degree of turnover determines the aroma quality. However, few studies have focused on fatty acid degradation in the whole course of oolong tea manufacture, especially during the green-making stage.

Fatty acids act as important aroma precursors of tea [12,13,14], and their constituents differ with tea locations, cultivars, maturity, harvest time, manufacturing technology, storage time, and so on [15,16,17,18,19]. As the maturity of fresh leaves increases, the contents of fatty acids increase, and their effect on tea quality also increases. Thus, the fresh leaves from oolong tea with relatively higher fatty acid contents should be given more attention, compared with green tea and black tea.

Currently, the composition of fatty acids and their derivatives from Tieguanyin tea and its fresh leaves have been characterized, but the quantitative accuracy needs to be further improved. Simultaneous distillation extraction (SDE) is preferred for qualitative volatile measurement [20,21,22]. However, this method suffered from artifacts due to its longer extraction time at relatively high temperatures. Unlike SDE, SAFE operated at relatively low temperature which preserves the volatile component profile [23,24], so we used this technique to study the aroma chemistry of Tieguanyin tea [25]. Moreover, fatty acids of oolong tea were determined by the internal standard method [18]. Currently, the dynamic variations of fatty acids during tea manufacture were undefined, and it was difficult to effectively improve aroma quality. In this study, the fresh leaves of Tieguanyin tea plant were prepared into oolong tea, and the processed samples were analyzed for fatty acids and FADV by the internal standard method, aiming to investigate the variations in fatty acids and FADV during oolong tea processing and further explore the relationship between fatty acids and key FADV.

## 2. Material and Methods

### 2.1. Chemicals and Reagents

Sulfuric acid, methanol, butylated hydroxytoluene (BHT), toluene, sodium chloride, anhydrous sodium sulfate, and diethyl ether were purchased from Sinopharm Chemical Reagent Co., Ltd. (Shanghai, China). Hexyl hexanoate, fatty acid methyl ester (FAME), and alkane reference standards were purchased from J&K Chemical Ltd. (Shanghai, China). Margaric acid and ethyl caprate were purchased from Sigma-Aldrich (Steinheim, Germany).

Thirty-seven FAME reference standards with concentrations between 100 ng/mL and 200 ng/mL (C4–C24) were mixed, while FAME reference standards with carbon numbers of C16-C18 were prepared individually, and the purity was 98–99%. The alkane reference standards (C4–C40) were dissolved in chloroform with a concentration of 500 μg/mL.

### 2.2. Preparation of Tea Samples

One bud and 3 leaves of Tieguanyin tea plants were plucked from tea fields at Fuqing Wuli Ecological Agriculture Science and Technology Co., Ltd. (Fuqing, China) in May 2018 and then manufactured into oolong tea and green tea (control). The manufacturing method of tea samples is described in the Supplementary Materials, Figure S1. Fresh leaves (FL) were withered or spread indoor with temperature of 21–23 °C. The green-making technology of oolong tea included 4 steps: the 1st turnover, 2nd turnover, 3rd turnover, and 4th turnover, the duration was 3 min, 4 min, 8 min, and 10 min, respectively. The interval between two successive turnover steps ranged from 2 to 3 h. After the completion of the 4th turnover/spreading cycle, the processed leaves were fixed in an electric-powered pan, the fixation was stopped once the leaf temperature reached 80–85 °C. Then, the fixed leaves were rolled for 35 min, followed by drying at 100 °C for 10 min and 90 °C for 1 h. Spreading leaves, green-making leaves, 1st turnover leaves, 2nd turnover leaves, 3rd turnover leaves, 4th turnover leaves, fixed leaves of oolong tea and green tea, and rolled leaves of oolong tea and green tea were frozen at −80 °C. Rolled leaves were dried and prepared as raw tea.

### 2.3. Extraction and Analysis of Fatty Acids

Tea samples except raw tea were ground into powder by a mortar and pestle containing liquid nitrogen, the tea powder was divided into two parts: one part was freeze dried to analyze fatty acids, and the other was prepared for aroma analysis. The method of FAME preparation was conducted as previously published [18]. Forty milligrams of the sample, 1 mL of 5% (*v*/*v*) sulfuric acid in methanol, 75 μg of internal standard heptadecanoic acid (C17:0), 300 μL of toluene, and 20 μL of BHT were put into an 8 mL glass tube and heated at 95 °C for 1.5 h. After heating, 1 mL of NaCl (0.9%, *m*/*v*) and 3 mL of hexane were added, mixed completely, centrifuged at 1500× *g* for 15 min, and then transferred to a new tube. A total of 3 mL of hexane was added and extracted again. The extracts were combined and dried with N_2_. The residue was resuspended in hexane and analyzed by GC–FID.

Fatty acids of the samples were analyzed by GC–FID as described previously [18] with slight modifications. One microliter of the sample was injected into a capillary column (DB-23 column, 60 m × 0.25 mm × 0.25 µm) in split mode. The flow rates of hydrogen, nitrogen, and zero air were 40 mL/min, 30 mL/min, and 400 mL/min, respectively. The split ratio was 10:1, the injection port temperature was 240 °C, and the detector temperature was 220 °C. The oven temperature was initially set at 100 °C and increased to 170 °C at a rate of 6.5 °C/min, where it was held for 12 min. Then, the temperature was increased to 230 °C at a rate of 4 °C/min and held for 3 min. Fatty acids were identified by comparing with FA standards and quantified by the internal standard method.

### 2.4. Extraction and Analysis of the Odorant Compounds

The volatile compounds of tea samples were extracted by the SAFE method. The sampling process was different depending on the processing step: fresh leaves, spreading leaves, and turnover leaves were weighed to 8 g; fixed leaves and rolling leaves were weighed to 6 g, and raw tea was weighed to 2 g. Tea samples, 40 mL of diethyl ether, 64.5 µg of internal standard ethyl caprate, and 1~8 g of anhydrous sodium sulfate were put into a Erlenmeyer flask and then extracted under stirring for 2 h. The organic phase was collected and then distilled through the SAFE apparatus. The detailed SAFE operation follows the reported method [25]. The volume for the distillation flask and the receiver flask was 500 mL. The distillate was collected, aliquoted into an 8 mL glass tube, concentrated in a rotary evaporator to approximately 1.5 mL, transferred into a GC vial, and further concentrated in a rotary evaporator. The volume was closely monitored; the concentration step was stopped immediately when the volume was slightly below the 500 µL mark. A small volume of diethyl ether was added to adjust volume to 500 µL. Fresh leaves and their processing samples had four biological replicates, whereas green tea samples had three biological replicates.

Volatile compounds were analyzed as described previously [26]. In brief, 1 μL of the sample was injected into a capillary column (RXi-5SiIMS column, 30 m × 0.25 mm × 0.25 µm) in spitless mode for GC–MS and GC–FID analysis. The oven temperature program was initiated at 50 °C for 2 min, raised 5 °C/min to 180 °C and held 2 min, and then raised 10 °C/min to 230 °C and held for 5 min before returning to 50 °C for the next sample. The injector, ion source, interface, and FID temperatures were 230 °C, 250 °C, 270 °C, and 230 °C, respectively. The flow rates of hydrogen, nitrogen, and air were 40 mL/min, 30 mL/min, and 400 mL/min, respectively, and helium was used as the carrier gas with flow rates of 1.0 mL/min and 1.7 mL/min for GC–MS and GC–FID, respectively. The electron impact ion source was set at 70 eV, and the chromatograms were recorded by monitoring the total ion current in the mass range of 45–600 *m*/*z*. Volatile compounds were identified from MS data by searching against the NIST14 library and authentic standards and quantified from FID data by normalizing to the internal standard peak area. In addition, the volatile compound content of the tea samples was calculated, deducting the water content analyzed by direct drying method (GB 5009.3-2017). 

### 2.5. Data Processing

All chemical analyses are expressed as the mean ± standard error (SE). Two-tailed Student’s *t*-test (T.TEST) was used to compare the difference between fresh leaves and raw tea. Correlation analysis (version 21.0, IBM Corp., Armonk, NY, USA) was conducted to explore the relevance of fatty acids and FADV during tea processing. The Origin software program (version 2018, Origin Lab Corp., Northampton, MA, USA) was used for graphing.

## 3. Results and Discussion

### 3.1. Change Properties of Fatty Acids during Tieguanyin Tea Processing

#### 3.1.1. Fatty Acid Composition and Content in Tieguanyin Tea Samples

To date, more than ten kinds of fatty acids have been detected in tea samples, and linolenic acid (C18:3) is predominant in fresh leaves of tea plants and prepared tea. Metabolites of fatty acids are important odorants of oolong tea. It is known that the degree of fatty acid degradation is related to tea manufacturing methods. Although changes in fatty acids during black tea manufacture have been studied [15], in semifermented tea such as oolong tea they remain unclear. To uncover the variations in fatty acid levels during oolong tea manufacturing, fresh Tieguanyin leaves were prepared into oolong tea and green tea with traditional technology. In Tieguanyin tea samples, 15 fatty acids were detected (Table 1), including 6 saturated fatty acids (SFA) and 9 unsaturated fatty acids (USFA). Among them, C12:0, C14:0, C16:1, C20:0, C20:2, C20:3, and C20:4 were below 100 μg/g, while C16:0, C18:0, C18:1, C18:2, C18:3, C20:1, C24:0, and C24:1 were over 100 μg/g. C18:3 was the most abundant one of all the fatty acids, and C16:0 was the highest saturated fatty acid in our samples, which differed from tea oil results [27,28]. 

**Figure 1:**
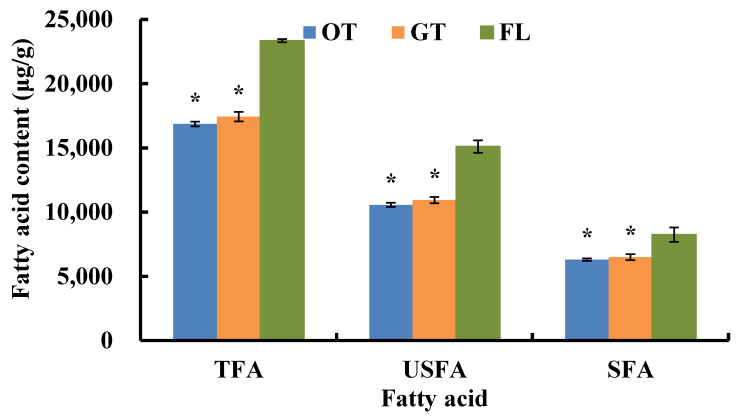
Fatty acids content of different tea samples. TFA, USFA, and SFA indicate total fatty acids, unsaturated fatty acids, and saturated fatty acids, respectively; * indicates the difference level of fatty acids between fresh leaves (FL) and oolong tea (OT) or green tea (GT) (*p* < 0.05).

Generally, the USFA content of tea leaves was higher than that of SFA, and the ratio of USFA to SFA changed with the processing method. We found that fatty acids decreased greatly after fresh leaves were turned into made tea (Figure 1), which was consistent with Guo et al. [18]. The total fatty acids (TFA), USFA, and SFA contents of raw tea were significantly lower than those of fresh leaves (*p* < 0.05), and oolong tea had lower fatty acids content than green tea. Namely, the manufacturing technology of oolong tea was relatively beneficial to fatty acid degradation, and different fatty acids showed variable degradation degree (Table 1). C12:0 contained the shortest carbon chain of the detected fatty acids, and its content decreased faster than that of C18:3 and C16:0. The C18:3 content of fresh leaves reached 9622.6 μg/g and decreased to 6734.1 μg/g in oolong tea, which accounts for a 29.0% reduction. C16:0 showed slower decreasing tendency and was reduced by 16.4% in oolong tea compared with C18:3. C20:4 degraded almost completely so that it was also undetected in Minbei oolong tea, possibly due to the length of the carbon chain and the number of double bonds [19]. Like C20:4, C18:3 degraded much more than others among fatty acids with 18 carbons. 

#### 3.1.2. Change in TFA, USFA, and SFA during Tieguanyin Tea Manufacturing

**Figure 2:**
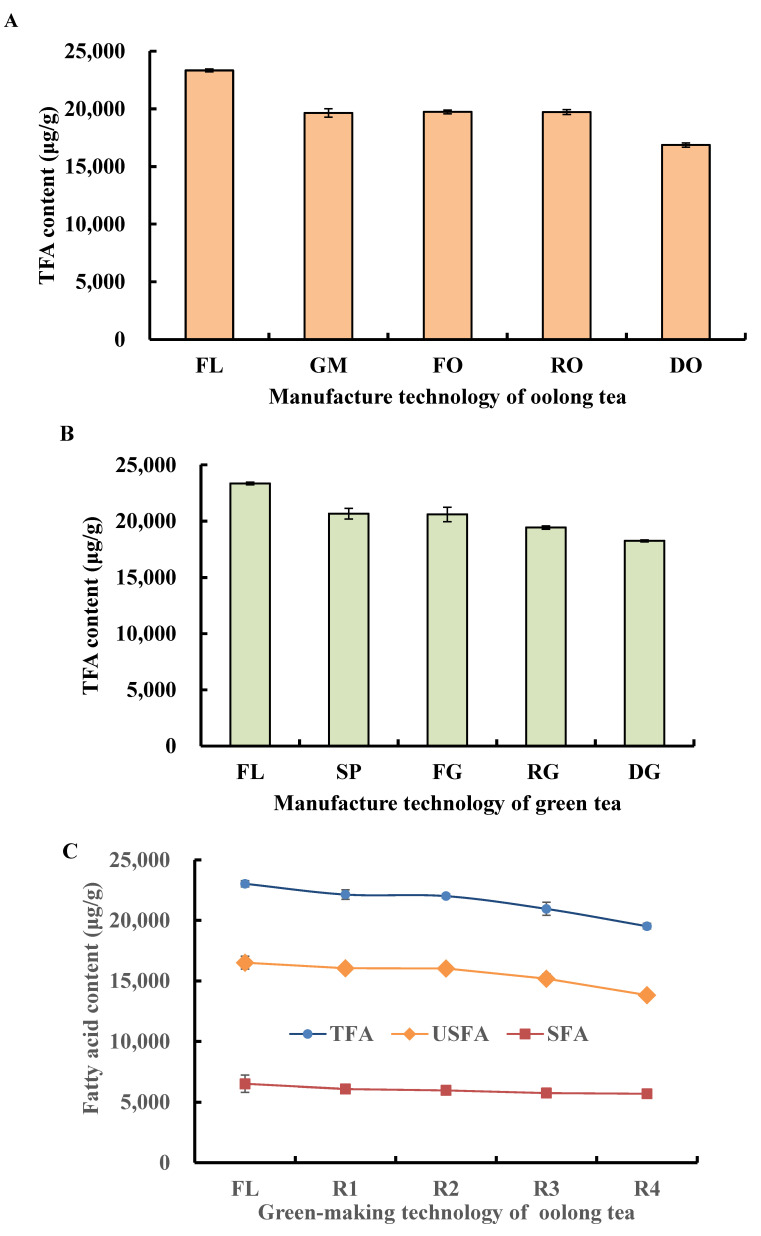
Fatty acids influenced by tea processing technology. (**A**) Total fatty acids changed during oolong tea processing, (**B**) total fatty acids changed during green tea processing, (**C**) different fatty acids changed during the green-making stage. TFA, USFA, and SFA indicate total fatty acids, unsaturated fatty acids, and saturated fatty acids, respectively; FL, GM, FO, RO, and DO indicate fresh leaves, green-making step, fixation of oolong tea, rolling of oolong tea, and drying of oolong tea, respectively; FL, SP, FG, RG, and DG indicate fresh leaves, spreading, fixation of green tea, rolling of green tea, and drying of green tea, respectively; and FL, R1, R2, R3, and R4 indicate fresh leaves, 1st turnover, 2nd turnover, 3rd turnover, and 4th turnover of oolong tea, respectively.

The tea manufacturing method influenced the composition of fatty acids. During oolong tea and green tea preparation, TFA declined continually (Figure 2A,B), and the spreading and green-making steps were the major points of fatty acid variation. The TFA content of GM leaves was 1021.3 μg/g lower than that of SP leaves; that is, green-making technology had a relatively larger effect on fatty acid degradation. Fixation and rolling technology had less influence than green-making technology; therefore, the TFA contents of fixation and rolling leaves were at the same level as that of GM leaves. Drying was the last manufacturing step and greatly reduced the content of fatty acids, which was beneficial to extend the shelf life of the tea. However, the degree of fatty acids conversion promoted by drying technology was less than by the green-making technology.

To further investigate the effect of green-making technology, the fatty acids were analyzed from processed samples. In this study, the green-making step of Tieguanyin tea was divided into four steps (R1, R2, R3, and R4), and previous studies have demonstrated that each step played different roles in aroma quality formation. R1 was the first turnover and represented a “uniformly rotated” state, and then subjected to the second turnover where the samples seemed lively again, that is, R2 showed a “lively rotated” state. After completion of the third and fourth turnover cycles, the processing samples showed a charming smell, and unique aroma characteristics started to emerge. Figure 2C showed that during the green-making stage, the contents of TFA, USFA, and SFA similarly decreased, and their contents were related to the number of turns. Compared with those in fresh leaves, the TFA, USFA, and SFA contents in R4 leaves declined by 15.2%, 16.3%, and 12.5%, respectively, while those in R1 leaves were least affected, and only decreased from 2.8% to 6.6%. The relative content of USFA decreased with every turnover to a greater degree than SFA, and the largest reduction appeared after the final turn. In R4 samples, the USFA and SFA contents decreased to 13,538.1 μg/g and 5689.4 μg/g, respectively. From this observation, USFA were determined to be the major fatty acids degraded during the green-making stage.

#### 3.1.3. Change in Fatty Acids Composition during Tieguanyin Tea Manufacturing

**Figure 3:**
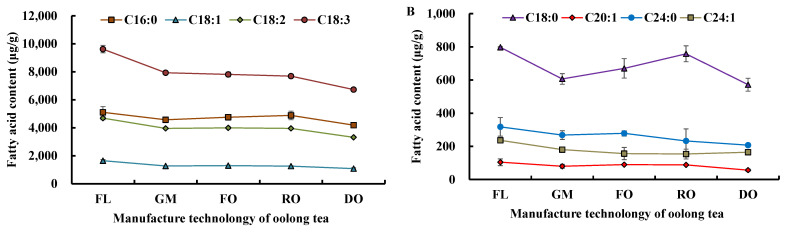

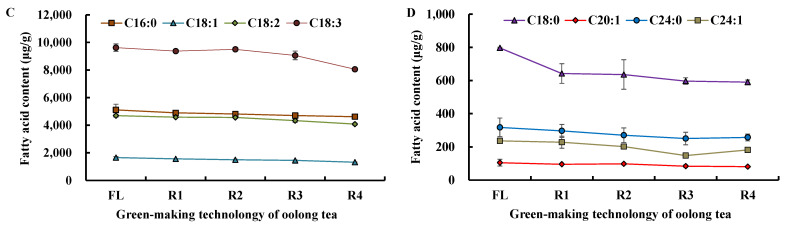
Manufacturing technology influence on fatty acids composition. (**A**) C16:1, C18:1, C18:2, and C18:3 changed during oolong tea making; (**B**) C18:0, C20:1, C24:0, and C24:1 changed during oolong tea making; (**C**) C16:1, C18:1, C18:2, and C18:3 changed during the green-making stage; and (**D**) C18:0, C20:1, C24:0, and C24:1 changed during the green-making stage. FL, GM, FO, RO, and DO indicate fresh leaves, green-making step, fixation of oolong tea, rolling of oolong tea, and drying of oolong tea, respectively; FL, SP, FG, RG, and DG indicate fresh leaves, spreading, fixation of green tea, rolling of green tea, and drying of green tea, respectively; and FL, R1, R2, R3, and R4 indicate fresh leaves, 1st turnover, 2nd turnover, 3rd turnover, and 4th turnover of oolong tea, respectively.

In our tea samples, the changes in fatty acids composition, except for C12:0, C14:0, C16:1, C20:0, C20:2, C20:3, and C20:4, were analyzed during oolong tea preparation (Figure 3A,B). All fatty acids content decreased obviously after the fresh leaves were prepared as the made tea, and their variations during oolong tea manufacture differed with chain lengths and double bond numbers (Figure 3A,B). C16:0 and C18:0 decreased at the green-making and drying steps, while they increased at the fixation and rolling steps. Similar to C16:0 and C18:0, C24:0 also has no double bonds, and its content was increased by fixation. The contents of C18:1 and C18:2 showed a similar tendency to that of C24:0. Although C18:3 has the same number of carbon atoms as C18:1 and C18:2, its content decreased gradually during oolong tea preparation. The effect of green-making technology on C18:3 was the strongest among all the fatty acids. 

During the green-making stage, the processed leaves undergo changes in response to the exposure of multiple stresses to minimize such adverse effects, similar to other plants [29]. We found that different fatty acids showed various trends at the green-making stage of oolong tea (Figure 3C,D). C16:0, C18:0, C18:1, and C18:2 decreased in abundance continuously; the contents of C18:3 and C20:1 increased after the second turnover; while the contents of C24:0 and C24:1 increased at the final turnover. Under adverse conditions, fatty acids in tea plants changed their structures and increased in unsaturation [30,31]; therefore, C18:3 and C20:1 increased in abundance from R1 to R2 and C24:0 and C24:1 increased in abundance at the final turnover, possibly resulting from long-chain fatty acid degradation.

Fatty acids varied with the turnover process, and their abundances also changed. At the first turnover, the contents of C18:0, C20:1, and C24:0 decreased most rapidly, and account for a 19.4%, 9.2%, and 6.7% reduction, respectively. C18:3 underwent the largest reduction. At the fourth turnover, the contents of C18:3, C18:1, and C18:2 decreased rapidly and became the fatty acids that decreased the most. Unexpectedly, the C18:3 content increased slightly at the second turnover, and the C24:1 content showed the fastest decline from the second to the third turnover, so the C24:1 content in R3 leaves was lower than that in R1, R2, and R4 leaves. Thus, fatty acid degradation was influenced by tea manufacturing technology, and the degree of degradation was related to chain length and desaturation levels of the fatty acids.

### 3.2. Change in FADV Content during Tieguanyin Tea Processing

During the process of turning fresh leaves into made tea, the fatty acids content decreased significantly, but only partial of the fatty acids were converted to FADV such as alcohols, esters, aldehydes, ketones, and hydrocarbons [13,14,32]. Total ion chromatogram of three samples was shown as a representative (Figure S2). In our Tieguanyin tea samples, 20 volatile compounds were detected which probably derived from fatty acid degradation (Tables S1 and S2), they were: (E)-3-hexenyl butyrate, (Z)-3-hexenyl butyrate, and hexyl butyrate. Based on the functional group, these FADV were divided into fatty acid esters, aromatic esters, jasmonates, and fatty alcohols. The FADV composition and content of tea samples differed with tea types and manufacturing steps.

#### 3.2.1. FADV Profiles and Contents in Tieguanyin Tea Samples

**Figure 4:**
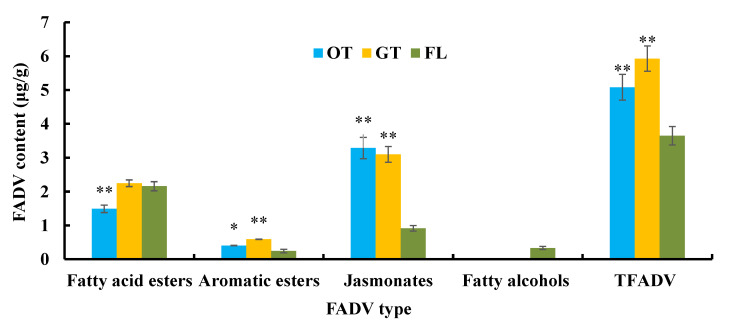
FADV content of different tea samples. TFADV indicates total fatty acid derivatives. * and ** indicate the difference between fresh leaves (FL) and oolong tea (OT) or green tea (GT) at *p* < 0.05 and *p* < 0.01, respectively.

The number of FADV in oolong tea and green tea was greater than in the fresh leaves, which proved that tea manufacturing technology promoted FADV production. Among the 11 FADV detected in oolong tea, the methyl jasmonate content (2.85 μg/g) was relatively high, and hexyl hexanoate, (E)-2-hexenyl hexanoate, cis-jasmine lactone, and (Z)-3-hexenyl octanoic acid were below 1.0 μg/g and undetectable from fresh leaves. Among the 15 FADV detected in green tea, (Z)-3-hexenyl butyrate, (Z)-3-hexenyl 2-methylbutyrate, hexyl 2-methyl butyrate, (E)-2-hexenyl benzoate, and cis-3-hexenyl salicylate were not found in the fresh leaves and oolong tea. Nonadecanol-1 and 1-ethoxypentan-3-ol were not found in oolong tea and green tea; that is, fatty alcohols were detected only in the fresh leaves (Figure 4).

After the fresh leaves were processed into raw tea, the FADV content changed. *t*-test analysis showed that total FADV (TFADV) content of raw tea was significantly higher than that of the fresh leaves (*p* < 0.01). Compared with green tea, oolong tea had a lower FADV content (5.08 μg/g), which was in discordance with the fatty acid variation. Except for methyl jasmonate, the contents of other FADV detected in oolong tea added up to 2.23 μg/g, while that in green tea reached 3.57 μg/g. We found that the fatty acid ester content in oolong tea was significantly lower than that in the fresh leaves (*p* < 0.01), but those in green tea and fresh leaves were similar. The aromatic ester contents in raw tea were higher than that in the fresh leaves, and the significance levels were *p* < 0.05 and *p* < 0.01, respectively. However, the contents of jasmonates with floral aromas in oolong tea and green tea were significantly higher than in fresh leaves (*p* < 0.01) and there was no difference between oolong tea and green tea. Thus, the difference in FADV content between oolong tea and green tea could be determined from their composition and content, resulting from different manufacturing technologies.

#### 3.2.2. Changes in FADV during Tieguanyin Tea Manufacturing

**Figure 5:**
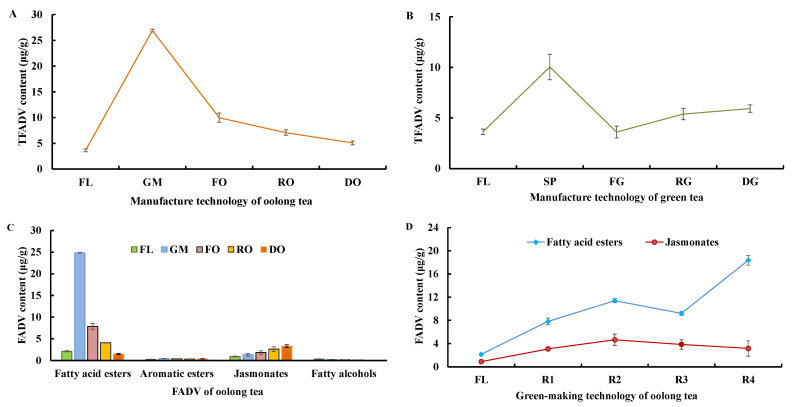
FADV influenced by tea processing technology. (**A**) Total FADV changed during oolong tea processing; (**B**) total FADV varied during green tea processing; (**C**) different FADV changed during oolong tea processing; and (**D**) the effect of green-making technology on fatty acid esters and jasmonates. TFADV indicates total fatty acid derivatives; FL, GM, FO, RO, and DO indicate fresh leaves, green-making step, fixation of oolong tea, rolling of oolong tea, and drying of oolong tea, respectively; FL, SP, FG, RG, and DG indicate fresh leaves, spreading, fixation of green tea, rolling of green tea, and drying of green tea, respectively; and FL, R1, R2, R3, and R4 indicate fresh leaves, 1st turnover, 2nd turnover, 3rd turnover, and 4th turnover of oolong tea, respectively.

During tea manufacturing, the TFADV content of samples varied with fatty acid reduction (Figure 5A,B); the green-making and spreading techniques showed greater effects on FADV than the other techniques, and new FADV such as 4-methylpentyl isobutyrate, (Z)-3-hexenyl butyrate, hexyl butyrate, (Z)-2-hexenyl butyrate, 2-methyl-(Z)-3-hexenyl butyrate, 2-methyl-hexyl butyrate, (E)-2-hexenyl isovalerate, hexyl hexanoate, (E)-2-hexenyl hexanoate ester, cis-jasmine lactone, and (Z)-3-hexenyl octanoic acid were produced. The TFADV content first increased and then decreased during oolong tea preparation, and the peak value appeared at GM and exceeded the SP leaves of green tea. The TFADV content of the GM sample (26.92 μg/g) was 2.7-fold, 3.8-fold, and 5.3-fold that of fixation, rolling and drying samples of oolong tea, respectively. The TFADV content increased at RG and remained at a lower level than RO; thus, the TFADV content of green tea was less than that of oolong tea.

Different types of FADV showed different changes during oolong tea preparation (Figure 5C), and only fatty acid esters demonstrated the same trend as TFADV. The percentage of fatty acid esters in TFADV ranged from 28.8 to 92.3% and reached a peak at the GM step. The content of fatty acid esters of the GM sample was 11.5-fold that of fresh leaves and then decreased to 1.48 μg/g in oolong tea. The content of jasmonates was less than that of fatty acid esters in the fresh leaves, which increased gradually and reached 3.28 μg/g in oolong tea. The contents of aromatic esters and fatty alcohols were relatively low and slightly changed, so we speculated that fatty acid esters and jasmonates probably played an important role in aroma quality formation.

The content of fatty acid esters and jasmonates varied as the number of turnovers increased (Figure 5D). The order of fatty acid ester content was fresh leaves (FL) < R1 < R3 < R2 < R4, indicating that the 3rd turnover was the key point of fatty acid ester variation. The fatty acid ester content of the R3 sample was 19.3% and 50.0% lower than those of R2 and R4, respectively. Jasmonates exhibited a different order: FL < R1 < R4 < R3 < R2, that is, the jasmonates content was more influenced by the fourth turnover. The jasmonates content of R4 leaves was 31.9% and 17.9% lower than those of R2 and R3, respectively. Further analysis revealed that unlike the fourth turnover, the first three turnovers had a similar effect on fatty acid esters and jasmonates (FL < R1 < R3 < R2).

### 3.3. Correlations of Fatty Acids and Their Derivatives during Oolong Tea Processing

**Figure 6:**
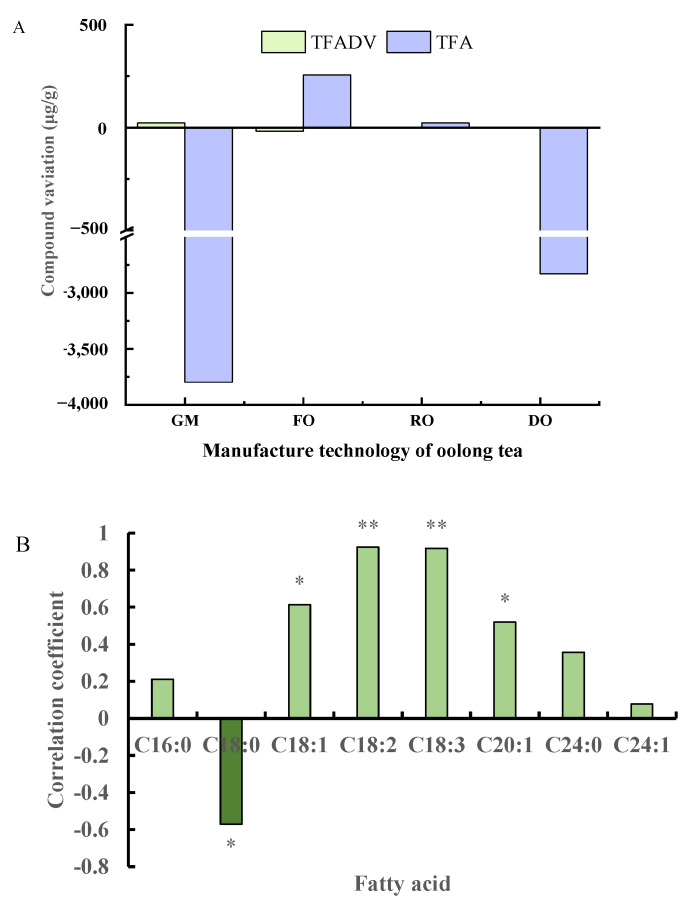
Relation in FA and FADV contents during oolong tea preparation. GM, FO, RO, and DO indicate the green-making step, fixation, rolling, and drying technology of oolong tea, respectively; (**A**) indicates the variation in fatty acids content and their volatile derivative contents differed with the manufacturing technology; and (**B**) indicates the relation between the key FAs and MeJA during the green-making stage of oolong tea. * and ** are the relationships of different FAs with MeJA at *p* < 0.05 and *p* < 0.01, respectively.

After fresh leaves were prepared as raw tea, the fatty acids content decreased to a minimum. The variation in fatty acids content and their volatile derivative contents differed with the manufacturing technology (Figure 6A). At the green-making and drying steps, the TFA content decreased greatly, while that of TFADV increased at the green-making step. Contrary to the variation in TFA content, the TFADV content increased at the fixing and rolling stages, but the absolute variations in TFADV and TFA contents were similar at the rolling step. Moreover, the variation in TFADV content was under 100 μg/g and far below that of the TFA content. Further analysis showed a significant and negative correlation between the fatty acids and their derivatives (R^2^ = −0.823, *p* < 0.05). Therefore, we can conclude that tea manufacturing technology had a stronger effect on fatty acid degradation than FADV production.

### 3.4. Relationship of the Key FADV with Fatty Acids during Oolong Tea Green-Making Stage

MeJA, the most important FADV, is a type of jasmonate with four isomers [33,34], which is only differentiated by nonpolar columns such as DB-5 and HP-5 columns. However, in addition to MeJA, other FADVs analyzed by nonpolar columns have become a good reference. In our samples, only one MeJA structure was identified as previously published, and its content increased in the whole course of oolong tea preparation (Supplementary Materials, Table S1). Feng et al. reported that when the MeJA content was over 0.15 μg/g [35], tea samples had orchid aromas, so MeJA could be selected as a representative FADV.

The fatty acids content changed more greatly during oolong tea preparation than during green tea preparation, contributing to FADV production, especially at the green-making stage. To date, the relationship of fatty acids with FADV has been analyzed in black tea [36], but the fermentation levels of oolong tea and black tea are different [37] and exhibit different variations. Pearson correlation was applied to analyze the variation in the contents of key fatty acids and MeJA during the oolong tea green-making stage (Figure 6B). The results showed that the relative levels of every fatty acid with MeJA were different, and the correlation coefficients ranged from −0.571 to 0.924. C18:1 and C20:1 showed a significantly positive correlation with MeJA (*p* < 0.05) and C18:2 and C18:3 showed an extremely significant positive correlation (*p* < 0.01); however, C18:0 was significantly and negatively correlated (*p* < 0.05). Among the eight fatty acids mentioned above, only C18:0 demonstrated a significant and negative correlation with MeJA, indicating that the degradation of SFA, including C16:0, C18:0, and C24:0, may provide less support for FADV formation than that of USFA. Therefore, USFA have potential as a new mark to regulate manufacturing methods.

## 4. Conclusions

In this research, we found that USFA were dominant and became the main degradation precursors during oolong tea manufacturing, especially at the green-making stage. The large impact of green-making technology on fatty acid levels occurred at the first and fourth turnovers, differing from FADV. Among the FADV, the contents of fatty acid esters and jasmonates were optimal so that the processing samples showed a floral characteristic after the first three turnovers. Through correlation analysis, we found that fatty acid degradation was beneficial to FADV formation during oolong tea processing. MeJA was a representative FADV and positively correlated with multiple fatty acids except for C18:0 at the green-making stage.

## Figures and Tables

**Table 1:** Fatty acids in Tieguanyin fresh leaves and raw tea.

Fatty Acid (Cm:n)	RT (min)	Fresh Leaves (μg/g)	Oolong Tea (μg/g)	Green Tea (μg/g)
C12:0	10.412	46.1 ± 31.4	33.2 ± 6.3	20.8 ± 5.7
C14:0	13.494	53.8 ± 12.7	48.3 ± 3.7	45.7 ± 1.7
C16:0	17.198	5105.5 ± 411.4	4192.8 ± 15.4	4267.4 ± 184.4
C16:1	17.854	59.2 ± 5.8	35.3 ± 2.3	188.1 ± 5.0
C18:0	21.279	796.9 ± 301.5	571.5 ± 38.8	615.5 ± 85.8
C18:1	21.906	241.1 ± 24.2	188.3 ± 39.7	155.1 ± 11.9
C18:2	23.009	4694.5 ± 111.0	3320.1 ± 45.8	3410.7 ± 85.5
C18:3	24.415	9622.6 ± 266.7	6734.1 ± 109.2	6833.0 ± 189.8
C20:0	25.600	57.8 ± 13.1	51.2 ± 10.7	46.9 ± 8.2
C20:1	26.320	104.5 ± 20.5	56.8 ± 23.7	72.0 ± 9.7
C20:2	27.760	38.8 ± 16.7	29.1 ± 5.6	22.6 ± 10.0
C20:4	29.314	43.3 ± 26.1	ND	54.0 ± 14.2
C20:3	29.701	57.8 ± 19.5	32.3 ± 7.5	27.4 ± 22.0
C24:0	38.738	396.2 ± 143.5	207.6 ± 10.0	285.2 ± 9.9
C24:1	39.838	236.3 ± 13.5	165.1 ± 6.0	173.3 ± 26.3

Note: m and n represent the number of carbon atoms and double bonds of each fatty acid, respectively; RT is the retention times; ND is not detected.

## Data Availability

Not applicable.

## Supplementary Materials

The following supporting information can be downloaded at: https://www.mdpi.com/article/10.3390/foods11111563/s1, Figure S1: Technological process of oolong tea and green tea; Figure S2: Total ion flows of the volatile components in oolong tea samples; Table S1: FADV contents and compositions of oolong tea samples; Table S2: FADV contents and compositions of green tea samples.
