# Supplementary material for: Variations in Fatty Acids Affected Their Derivative Volatiles during Tieguanyin Tea Processing
Source: Foods. 2022 May 26;11(11):1563. doi: 10.3390/foods11111563 (PMC9180273; doi:10.3390/foods11111563)
Supplement: Supplementary file 1 [file foods-11-01563-s001.zip › foods-1721035-supplementary.pdf]

foods

MDPI

Supplementary Materials

Variations in Fatty Acids Affected Their Derivative Volatiles during Tieguanyin Tea Processing

Li Guo [1], Mingjie Chen [2,*], Yaling Guo [3] and Zhi Lin [1,*]

[1] Tea Research Institute, Chinese Academy of Agricultural Sciences, Hangzhou 310008, China; guoli@tricaas.com
[2] College of Life Sciences, Henan Key Laboratory of Tea Plant Biology, Xinyang Normal University, Xinyang 464000, China
[3] College of Horticulture, Fujian Agriculture and Forestry University, Fuzhou 350002, China; yaling7819@126.com
* Correspondence: mjchen@xynu.edu.cn (M.C.); linz@tricaas.com (Z.L.)

Table S1. FADV contents and compositions of oolong tea samples.

Compounds	Retention time (min)	FL (μg/g)	GM (μg/g)	FO (μg/g)	RO (μg/g)	DO (μg/g)
(E)-3-hexenyl butyrate	13.190	0.57±0.06	0.41 ± 0.00	0.21 ± 0.06	nd	0.15 ± 0.02
4-methylpentyl isobutyrate	13.370	nd	10.43 ± 0.02	3.78 ± 0.54	nd	nd
(Z)-3-hexenyl butyrate	14.606	nd	1.38 ± 0.07	0.53 ± 0.32	0.58 ± 0.02	nd
hexyl butyrate	14.781	nd	0.23 ± 0.03	0.20 ± 0.13	0.41 ± 0.02	nd
(Z)-2-hexenyl butyrate	14.862	nd	3.19 ± 0.00	0.34 ± 0.03	0.18 ± 0.01	nd
(Z)-3-hexenyl 2-methylbutyrate	15.642	nd	0.10 ± 0.00	nd	nd	nd
hexyl 2-methylbutyrate	15.803	nd	0.07 ± 0.00	0.17 ± 0.04	nd	nd
(E)-2-hexenyl 3-methyl butyrate	15.900	nd	0.25 ± 0.01	0.15 ± 0.02	nd	nd
(Z)-3-hexenyl hexanoate	20.517	1.44 ± 0.09	4.68 ± 0.00	1.16 ± 0.10	1.22 ± 0.02	0.39 ± 0.02
hexyl hexanoate	20.671	nd	3.16 ± 0.01	1.17 ± 0.09	1.26 ± 0.03	0.37 ± 0.02
(E)-2-hexenyl hexanoate	20.745	nd	0.82 ± 0.01	0.30 ± 0.02	0.32 ± 0.00	0.22 ± 0.03
cis-jasmone	21.126	0.21 ± 0.01	0.20 ± 0.03	0.37 ± 0.07	0.25 ± 0.01	0.35 ± 0.04
nonadecanol-1	21.310	0.08 ± 0.02	nd	nd	nd	nd
cis-jasmin lactone	23.374	nd	0.30 ± 0.00	0.15 ± 0.00	0.15 ± 0.02	0.09 ± 0.01
1-ethoxypentan-3-ol	24.315	0.25 ± 0.02	0.26 ± 0.00	0.16 ± 0.02	0.14 ± 0.00	nd
(Z)-3-hexenyl octanoic acid	25.773	nd	nd	nd	nd	0.26 ± 0.01
(Z)-3-hexen-1-ol benzoate	25.954	0.11 ± 0.04	0.32 ± 0.02	0.17 ± 0.02	0.20 ± 0.01	0.15 ± 0.01
hexyl benzoic acid	26.293	0.13 ± 0.02	0.14 ± 0.00	0.23 ± 0.03	0.16 ± 0.01	0.26 ± 0.01
methyl jasmonate	27.041	0.70 ± 0.07	0.85 ± 0.33	1.77 ± 0.37	2.21 ± 0.50	2.85 ± 0.28
methyl palmitate	32.975	0.15 ± 0.01	0.14 ± 0.01	0.09 ± 0.01	0.10 ± 0.01	0.10 ± 0.04
Total		3.65 ± 0.27	26.92 ± 0.27	9.99 ± 0.91	7.08 ± 0.52	5.08 ± 0.38*

Table S2. FADV contents and compositions of green tea samples.

Compounds	Retention time (min)	FL (μg/g)	SP (μg/g)	FG (μg/g)	RG (μg/g)	DG (μg/g)
(E)-3-hexenyl butyrate	13.190	0.57 ± 0.06	nd	nd	nd	nd
(Z)-3-hexenyl butyrate	14.606	nd	nd	nd	nd	0.52 ± 0.01
butyrate, 2-methyl-, 3-hexenyl ester, (Z)-	15.642	nd	nd	nd	nd	0.12 ± 0.02
hexyl 2-methylbutyrate	15.803	nd	nd	nd	nd	0.08 ± 0.07
(Z)-3-hexenyl hexanoate	20.517	1.44 ± 0.09	2.88 ± 0.12	1.16 ± 0.05	1.24 ± 0.03	0.53 ± 0.01
hexyl hexanoate	20.671	nd	0.28 ± 0.01	0.54 ± 0.02	0.55 ± 0.02	0.43 ± 0.06
(E)-2-hexenyl hexanoate	20.745	nd	0.66 ± 0.04	0.37 ± 0.01	0.38 ± 0.01	0.10 ± 0.02
cis-jasmone	21.126	0.21 ± 0.01	0.22 ± 0.01	0.26 ± 0.12	0.36 ± 0.06	0.35 ± 0.04
n-nonadecanol-1	21.310	0.08 ± 0.02	nd	nd	nd	nd
cis-jasmin lactone	23.374	nd	nd	nd	nd	0.39 ± 0.29
1-ethoxypentan-3-ol	24.315	0.25 ± 0.02	0.31 ± 0.02	0.18 ± 0.01	0.24 ± 0.03	nd
(Z)-3-hexenyl octanoic acid	25.773	nd	nd	nd	nd	0.29 ± 0.02
(Z)-3-hexen-1-ol benzoate	25.954	0.11 ± 0.04	0.10 ± 0.01	0.09 ± 0.01	0.11 ± 0.02	0.16 ± 0.01
hexyl benzoic acid	26.293	0.13 ± 0.02	0.13 ± 0.00	0.13 ± 0.08	0.20 ± 0.03	0.21 ± 0.01
(E)-2-hexenyl benzoate	26.669	nd	nd	nd	nd	0.08 ± 0.00
methyl jasmonate	27.041	0.70 ± 0.07	1.15 ± 0.18	0.69 ± 0.25	2.08 ± 0.36	2.36 ± 0.01
cis-3-hexenyl salicylate	27.677	nd	nd	nd	nd	0.14 ± 0.01
methyl palmitate	32.975	0.15 ± 0.01	4.31 ± 0.87	0.19 ± 0.03	0.24 ± 0.01	0.18 ± 0.02
Total		3.65 ± 0.27	10.04 ± 1.26	3.61 ± 0.58	5.39 ± 0.56	5.93 ± 0.37*

Note: "nd" is undetected; FL, GM, FO, RO, DO, SP, FG, RG, and DG indicate fresh leaves, green-making step, fixation of oolong tea, rolling of oolong tea, drying of oolong tea, spreading, fixation of green tea, rolling of green tea, and drying of green tea, respectively.

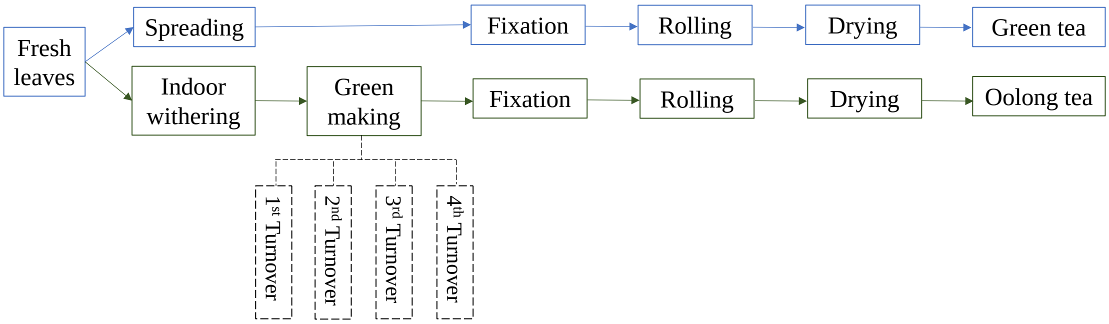

Figure S1. Technological process of oolong tea and green tea.

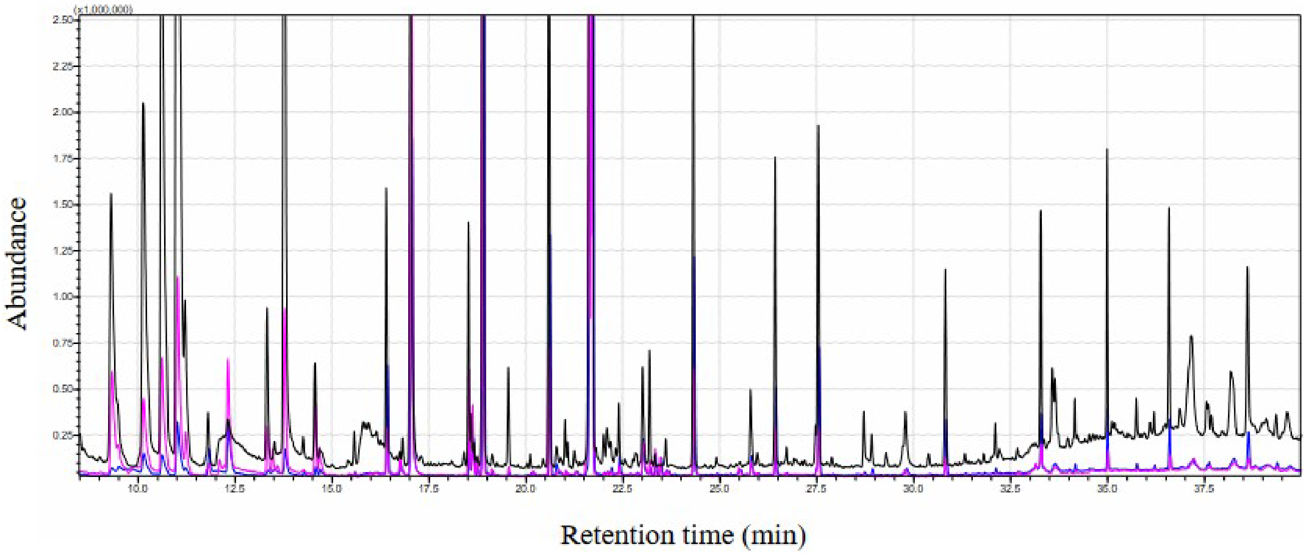

Figure S2. Total ion flows of the volatile components in oolong tea samples. Black line, purple line and blue line indicated total ion flows of sample FL, GM and RR, respectively.
